# Examining the impact of WHO’s Focused Antenatal Care policy on early access, underutilisation and quality of antenatal care services in Malawi: a retrospective study

**DOI:** 10.1186/s12913-019-4130-1

**Published:** 2019-05-08

**Authors:** Martina Mchenga, Ronelle Burger, Dieter von Fintel

**Affiliations:** 1 Faculty of Economics and management sciences, Stellenbosch University, Stellenbosch, South Africa; 2 Economics Department, Stellenbosch University, Stellenbosch, South Africa

**Keywords:** World Health organization (WHO), Focused antenatal care (FANC), 2016 ANC WHO guidelines, Quality, Early access, Underutilization, Malawi

## Abstract

**Background:**

A variety of antenatal care models have been implemented in low and middle-income countries over the past decades, as proposed by the World Health Organisation (WHO). One such model is the 2001 Focused Antenatal Care (FANC) programme. FANC recommended a minimum of four visits for women with uncomplicated pregnancies and emphasised quality of care to improve both maternal and neonatal outcomes. Malawi adopted FANC in 2003, however, up to now no study has been done to analyse the model’s performance with regards to antenatal care service quality and utilisation patterns.

**Methods:**

The paper is based on data pooled from three comparable nationally representative Malawi Demographic and Health Survey (MDHS) datasets (2000, 2004 and 2010). The DHS collects data on demographics, socio-economic indicators, antenatal care, and the fertility history of reproductive women aged between 15 and 49. We pooled a sample of 8545 women who had a live birth in the last 5 years prior to each survey. We measure the impact of FANC on early access to care, underutilisation of care and quality of care with interrupted time series analysis. This method enables us to track changes in both levels and the trends of our outcome variables.

**Results:**

We find that FANC is associated with earlier access to care. However, it has also been associated with unintended increases in underutilisation. We see no change in the quality of ANC services.

**Conclusion:**

In light of the WHO 2016 ANC guidelines, which recommend an increase of visits to eight, these results are important. Given that we find underutilisation when the benchmark is set at four visits, eight visits are unlikely to be feasible in low-resource settings.

## Background

A variety of antenatal care models have been implemented in low and middle-income countries over the past decades to improve maternal and child health outcomes, as proposed by the World Health Organisation (WHO) [1]. One such model is Focused Antenatal Care (FANC) programme. In this paper, we consider whether FANC contributed towards maternal health by improving early access, increasing the number of visits and enhancing quality. The WHO began promoting FANC in 2001, replacing the traditional antenatal care (ANC) service model which included numerous ANC visits (7–16 visits), and was a challenge in resource-constrained settings [2].

FANC recommends only four ANC visits for women with uncomplicated pregnancies and more otherwise. The four-visit model emphasises on quality of care and provides a package of services that contributes to the health and well-being of women throughout pregnancy, childbirth and the post-natal period [2]. The four visits in the WHO FANC model are scheduled to be made at specific times as follows: the first visit should occur between 8 to 12 weeks after conception but not later than 16 weeks; and, a further three visits should occur between 24 and 38 weeks of gestation [2].

Most low and middle-income countries, including Malawi, incorporated FANC into their health systems [1]. Despite the WHO revision of ANC guidelines to increase number of visits to eight in 2016, FANC is the ANC model currently in practice in Malawi [3]. Evidence shows that the model had substantial public health implications especially in low-income countries where healthcare resources are inadequate [1]. Moreover, in the 2015 Cochrane review [4], it was argued that the reduced visit model curtails the costs for women. This includes travel times to and from clinics, waiting time, transport costs where clinics are located far away, loss of working hours, and care of other children at home [4]. However, little is known about the impact of FANC on early access, utilisation and quality of ANC services in sub-Saharan African countries - including Malawi [5]. This study seeks to fill this research gap.

Previous research on the impact of FANC has been inconclusive. The WHO 2001 Trials conducted in Argentina, Cuba, Saudi Arabia and Thailand showed that FANC is safe and a sustainable, comprehensive and cost effective ANC model [2]. In Kenya, the adoption of FANC led to improved detection of existing diseases in pregnancy during the first ANC visit, planning for birth, prevention of complications and postpartum counselling [6]. In Ghana, FANC resulted in improved quality and continuity of care [7]. There are exceptions, however. In South Africa, FANC had no significant effect on the quality of ANC services. This was attributed to a lack of training, high staff turnover and inadequate supervision [8]. In the 2015 Cochrane review study, results showed that women in both low and high income settings were less satisfied with the reduced visit schedule and perceived the gap between visits as too long [4].

In Malawi, there is tentative evidence, based on research at one site that shows that the introduction of FANC led to improvements in the quality of ANC services at the facility [9]. Since the study used only one urban clinic, its external validity is questionable. Our study expands on this work by considering a nationally representative sample of women who accessed ANC in clinics across Malawi, including rural clinics, to assess the effectiveness of FANC at a national level.

Our study also adds to the literature on the impact of FANC in African countries by incorporating a time dimension in the analysis. We pool three cross-sectional Demographic and Healthy Survey (DHS) datasets and use the year of mother’s delivery as the date stamp (instead of survey year, see more details in Table 1). This is unlike previous studies that use one cross-sectional study at a point in time to look at correlations and therefore are not suited to provide statistical evidence on the effectiveness of FANC policy. Furthermore, our interrupted time series methodology allow us to track changes in both levels and trends of our outcome variables.

**Table 1:** Cross tabulation of birth year and FANC

DHS Data enumeration year	Woman’s year of delivery	FANC	Frequency
2000	1995	0	102
2000	1996	0	512
2000	1997	0	1023
2000	1998	0	1860
2000	1999	0	2494
2000	2000	0	2386
2004/2005	2001	0	791
2004/2005	2002	0	1352
2004/2005	2003	1	2308
2004/2005	2004	1	2240
2004/2005	2005	1	428
2010	2006	1	1348
2010	2007	1	2247
2010	2008	1	3545
2010	2009	1	3924
2010	2010	1	2203
Total sample size			28,763

Source: (DHS 2000, 2004 and 2010): 0, FANC policy not adopted: 1, FANC policy adopted

Malawi is also an important case study because of its challenging policy implementation environment due to high levels of maternal mortality [10], high levels of poverty [11], a lack of skilled medical personnel [12] and a lack of health infrastructure [9, 11]. In such resource-constrained settings, it is important to understand whether a reduction in the number of visits and stricter guidelines about the content of each visit could improve the quality of care and thus enhance maternal and child health. In this way, we are engaging with the debate on the appropriate model of care and the recently proposed WHO reforms to increase the recommended ANC visits to eight.

### Study context

Malawi is classified as a low-income country with a Gross Domestic Product (GDP) per capita as low as US$274 in 2014. This translates into US$0.75 to spend per day for the average individual [11]. Given the low GDP, the government has a limited tax base and faces dramatic trade-offs in its policy decisions while faced with considerable need [13]. These challenges are further exacerbated by a health system with poor infrastructure, lack of equipment and qualified human resources and weak management [12].

The Malawian healthcare delivery system is a three-tier system. The lowest level of care is the primary healthcare or community care system, which consists of community initiatives (village clinics), health posts, dispensaries, maternity units, health centres, community and rural hospitals. District hospitals constitute the secondary level and provide specialised services to patients referred from the primary healthcare level through outpatient and inpatient services and community health services. These services are enhanced by the provision of specialised supportive services, such as laboratory, diagnostic, blood bank, rehabilitation and physiotherapy services. Finally, tertiary healthcare consists of highly specialised services, and is provided by central and other specialist hospitals. These different levels are linked through an elaborate referral system [14].

Only 65% of facilities in Malawi offer antenatal care services [15]. A range of providers offers ANC, including government, non-profit and private providers [15]. In government facilities, the provision of ANC services is integrated with under-five clinics, family planning, postnatal care and other reproductive health services and it is provided free of charge [15]. For-profit and non-profit providers (such as the Christian Health Association of Malawi) require user fees at the point of use. As of 2010, 73% of ANC services were provided at primary health facility level on a daily basis while 27% were provided at secondary and tertiary levels [15]. In Malawi, nurses and midwives are the main providers of antenatal care services (80%), with the rest receiving care from doctors, clinical officers, or maternal-child health aides [15].

According to a Ministry of health report [5], Malawi adopted the FANC policy in 2003. FANC requires health facilities to have adequate infrastructure, clinical skills, essential equipment, drugs and laboratory supplies [9]. However, the government of Malawi did not take necessary measures to invest in the resources required for successful implementation of FANC. In 2010, annual monitoring results on the implementation of FANC showed that only one of the four central hospitals and four of the 24 district hospitals in Malawi met the WHO standards for delivering FANC [16]. Moreover, as of 2014, WHO reported that for every 10,000 people, there were only 0.2 doctors and 3.4 nurses and midwives in Malawi [12].

## Methods

### Data

The study is a retrospective study, which uses three Malawi Demographic and Health Survey (MDHS) datasets conducted in 2000, 2004/2005 and 2010. We chose the 3 years based on comparability. The MDHS provides detailed health information for women of reproductive ages between 15 and 49 and their children. The survey uses a multi-stage cluster sampling design to select households for participation based on the Malawi Population censuses of 1998 and 2008. The first stage comprised a random sampling of the enumeration areas and household listing operation followed by a random sampling of the households in the second stage. This study uses the women data file of the MDHS, which contains data on, among others: demographics, household socio-economic status, maternal health care and ANC utilisation practices. Information on ANC services utilisation and components of care is reported on women of reproductive ages 15–49 who had a live birth during the 5 years before each survey. The response rate for each survey was above 95%.

The main independent variable of interest in this study is the FANC policy dummy, which captures the year when FANC was implemented in Malawi (see Table 1 for details). We created the FANC policy dummy using mother’s year of delivery or the child’s year of birth. All women who gave birth after 2003 were categorised to be in the post-FANC period and those who delivered prior to 2003 are in the pre-FANC period. Our total initial pooled sample was 28,763 women; however, we limited the analysis to women who delivered 3 years before and after FANC adoption, excluding women who gave birth in 2003. This restriction reduces the final sample to 8545 women. Limiting the analysis to the years that were closest to the launch of the policy enabled us to avoid the influence of policies introduced prior to 2000 or after 2006, including the 2007 traditional birth attendant (TBA) ban [5].

### Main Outcome Measures

Our research considers the impact of FANC on three outcomes: early access, inadequate use/ underutilisation and quality of care.

### Early access to care

In this study, we use timing of first ANC visit to measure early access. This variable ranges from 0 to 9 months. A Weibull hazard model (an example of a survival-analysis model) was used to model the gestational age at which the mother enters the ANC system. Weibull models were initially developed to consider the survival of machine components, so that interpretation is sometimes counterintuitive: a higher likelihood of earlier ‘component failure’ here is equivalent to a higher likelihood of an ANC visit at an earlier gestational age. Survival-analysis models are common in the health sciences [17–19]. We study differences in early access to care before and after FANC was implemented.

### Underutilisation of care

FANC recommends a minimum of four ANC visits for women with uncomplicated pregnancies, therefore, in this context, a woman with less than the minimum number of four visits has underutilised the services.

For our model, we sought a more precise definition of underutilisation that does not overlap with that of early access. We therefore limit the sample to women who initiated their first ANC visit by 16 weeks of pregnancy as required by FANC to avoid duplication and overlap with the early access indicator. By limiting the analysis to women who accessed ANC early, we avoid a ‘double count’ problem where underutilisation may merely be another manifestation of late access. The definition also excludes non-users, who are captured in the early access indicator.

Underutilisation is a binary variable defined as 1 if a pregnant woman initiated her first visit in the first trimester of pregnancy but did not make the recommended number of four visits, and 0 otherwise. For the model, we considered cluster averages of underutilisation for the subset of women who initiated care early. Underutilisation is thus the likelihood of women in a specific cluster underutilising ANC service provided that they had a first visit as prescribed by FANC. We analyse this outcome with Ordinary Least Squares (OLS) regressions.

### Quality of care

The aim of FANC is not only to achieve a minimum number of four visits, but also compliance with FANC protocols. We therefore also track whether health care workers complied in conducting eight key ANC tests or examinations. These include routinely conducted diagnostics (taking blood and urine samples), physical examination (measuring blood pressure and weight), and other preventive procedures (administration of tetanus toxoid, prophylaxis, iron and folic supplements and establishing complication readiness). These questions were asked in each of the DHSes and are here interpreted as proximate indicators of the quality of ANC services.

As was the case for underutilisation, the analysis sample is also limited to women who initiated ANC early. The first visit recommended by FANC is critical for HIV infected pregnant women, to ensure access to antiretroviral (ARV) prophylaxis. In addition, pregnant women can access early interventions such as syphilis screening and treatment, provision of ferrous iron supplements and malaria prevention and treatment [20]. When a woman accesses her first ANC visit later than 16 weeks of pregnancy, she risks missing some of the early ANC interventions crucial for early detection of pregnancy complications. In such a case, missing some required interventions would therefore not represent non-compliance with FANC protocols, but result from late access.

Using the identified eight key measures, an ANC composite-compliance index was constructed using multiple correspondence analysis (MCA) [21, 22]. Table 2 lists the variables underpinning the composite index, with categories and weights for each variable. The weights are identified from the first dimension of the MCA. This dimension explained about 85% of the total inertia. Positive (negative) weights reflect the higher (lower) quality of care.

**Table 2:** Results for multiple correspondence analysis for the ANC quality index

Variable	Categories	Weights
Prophylaxis	Given malaria prophylaxis	0.417
	Not given malaria prophylaxis	−2.103
Blood pressure	Blood pressure measured	0.597
	Blood pressure not measured	−2.662
Blood sample	Blood sample taken and tested for disease	1.080
	Blood sample not taken or tested	−1.576
Urine sample tested	Urine sample taken and tested	1.927
	Urine sample not taken or tested	−0.557
Iron tablets	Iron tablets/syrup given	0.325
	Not given iron tablets/syrup	−1.789
Weight measured	Weight measured during visit	0.174
	Weight not measured	−5.196
Complications	Told about complications	0.514
	Not told about complications	−1.612
Tetanus toxoid	Given at least one tetanus toxoid vaccine	0.265
	Not given any tetanus toxoid vaccine	−1.686

The ANC-compliance index was then used as the main outcome variable in our OLS regression to estimate the statistical impact of FANC on quality of ANC services. Again, we aggregate at the community level. This shifts the attention from the demand side (women’s individual experiences) to the supply side (what we can expect a community clinic to provide on aggregate).

### Distinguishing FANC from confounding influences over this period

We are concerned about distinguishing the effects of FANC from two other shifts that occurred during this period: the expansion of education; the rise in HIV prevalence and launch of the prevention of mother-to-child transmission of HIV (PMTCT).

#### Woman’s education level

Women’s education level has been included as a categorical variable ranging from 0 to 3 (0, no education; 1, primary education; 2, secondary education and 3, tertiary education). Free primary education (FPE) in Malawi was implemented in 1994 [23]. We expect that post-FANC mothers are likely to be more educated than pre-FANC mothers. Higher educational attainment is associated with a higher propensity to access maternal health services [24, 25], thus it is important to include educational attainment in our models.

#### HIV testing and the introduction of PMTCT

In order to reduce HIV/AIDS prevalence, Malawi launched the national PMTCT programme in 2001 [26]. We are concerned that the promotion of PMTCT may have caused women to access care earlier and could have affected the quality of these services. To distinguish the impact of FANC from that of the PMTCT programme we include the community-level proportion of pregnant women who reported ever taking an HIV test.

### Other control variables

#### Pregnancy risk factors

As per FANC guidelines [2], women with uncomplicated pregnancies are recommended to make four ANC visits. However, women with a history of any complications or illnesses in previous pregnancies are identified to be high risk and recommended to make more than four visits. In this study, we include the following variables as proxies for a risky pregnancy: age at birth of less than 16 and more than 40, a history of miscarriage and/or caesarean deliveries. These indicators were chosen based on FANC guidelines [2] and data availability.

#### Type of health worker

In the analysis of the impact of FANC on quality of care, we also control for the type of health worker providing the ANC service to the client. We expect skilled health care providers such as doctors and nurses to be more knowledgeable and equipped to provide quality level service than a ward attendant. Studies in Nigeria [27] and Nepal [28] have reported that skills acquired by ANC providers have positive significant impacts on the quality of ANC offered. We define this variable as a series of binary variables (Doctor; nurse; TBA and health surveillance assistant/ward attendant), as captured in the DHS dataset. However, we exclude one category “other” to avoid the dummy variable trap.

#### Other factors

We expect rural based women to have poor access to clinics due to the longer travel distance to the closest facility. We also expect women who are exposed to media (such as radio or television) to have more health-related information and therefore to utilise ANC services more regularly. Other studies have found that media penetration through radio is important in influencing maternal healthcare utilisation [29, 30]. We can unfortunately only capture these traditional forms of media access, as the MDHS did not include questions on social media or internet access. It is however encouraging that supplementary statistics^1^ lead us to believe that at the time of the intervention, this was not an important channel through which FANC could have been promoted. Therefore, should not be an important channel of influence for FANC services uptake over the period of our analysis. By contrast, we see that close to half of the women in our sample say that they listen to the radio every day and radio was part of the government’s promotion campaign for FANC [5].

### Estimating the impact of FANC

We used interrupted time series analysis (ITSA) applied to OLS or Weibull models to estimate the changes in the outcome variables of interest between the two periods. ITSA allows us to capture both changes in levels and changes in trends. The design is particularly suited to interventions introduced at a population level over a clearly defined time period that target population-level health outcomes [32–34]. In standard ITSA, the following segmented-regression model is used:

1 $$ {Y}_{it}={\beta}_0+{\beta}_1{T}_{it}+{\beta}_2{\mathrm{X}}_{it}+{\beta}_3{T}_{it}{X}_{it}+{\beta}_4{Z}_{it}+{\mu}_{it} $$

Where *Y*_*it*_ is one of the outcomes of interest (in our case, quality of ANC services index; timing of first ANC visit; proportion of women with inadequate visits/underutilisation). *i* indexes either the individual or geographic cluster. *t* indexes time or year of delivery. *β*_0_ represents the baseline level of the outcome at *t* = 0, *β*_1_ is interpreted as the change in outcome associated with a time-unit increase (representing the underlying pre-intervention trend), *β*_2_ captures any discrete changes following the intervention,  *X* is the FANC dummy variable indicating the pre-intervention period (coded 0) or the post-intervention period (coded 1). *T* measures the time before (negative) or after (positive) the intervention, and is included as a linear trend. *β*_3_ indicates the slope change following the intervention (using the interaction between time and intervention: *TX*). *Z* is a vector of controls for confounding factors and *μ* is the error term. Our interest lies in assessing whether the time progression has been altered by FANC, so that is our coefficient of interest. All statistical analyses were conducted in Stata 14.

## Results

### Descriptive analysis

Descriptive statistics in Table 3 illustrate changes between the pre- and post-FANC periods in some of the variables used in our analysis. The results show significant increases in early access and quality of ANC services in the post-FANC period. However, the average number of ANC visits declined to just below the minimum number of four visits required by FANC in the post-FANC period.

**Table 3:** Social and demographic characteristics

Variables	Means or Proportions		*P*-value
	Pre- FANC	Post- FANC	Difference in means or proportions
Main outcome variables			
Early access	0.26	0.29	0.0007^***^
Number of visits	4.04	3.76	0.0000^***^
Standardized ANC quality index	0.71	0.74	0.0000^***^
Confounding variables			
Mother’s education level			
No education	0.27	0.21	0.0000^***^
Primary	0.62	0.65	0.0369^**^
Secondary	0.10	0.14	0.0000^***^
Tertiary	0.00	0.01	0.0291^**^
Type of residence			
Rural	0.85	0.84	0.0013^**^
Urban	0.15	0.14	0.0013^**^
HIV-test statistics			
Women who reported having ever been tested for HIV/AIDS	0.15	0.41	0.0000^***^
Pregnancy risk indicators			
Age: 16 and below	0.02	0.02	0.6901
Age: above 40	0.04	0.05	0.1182
History of miscarriages	0.11	0.13	0.0059^*^
History of caesarean deliveries	0.03	0.05	0.0004^***^
Media access			
Radio			
Not at all	0.23	0.22	0.3416
Less than once a week	0.18	0.16	0.0014^**^
At least once a week	0.12	0.16	0.0000^***^
Almost every day	0.47	0.46	0.2414
Television			
Not at all	0.90	0.83	0.0000^***^
Less than once a week	0.05	0.08	0.0000^***^
At least once a week	0.02	0.03	0.0000^***^
Almost every day	0.03	0.06	0.0000^***^

Notes: ^***^1% level of significance; ^**^ 5% level of significance and ^*^10% level of significance

The proportion of women with no education is lower in the post FANC period at 21% compared to the pre-FANC period at 27%. The proportion of women who reported having been tested for HIV between the two periods jumped from 15% before FANC to 41% after FANC. We see a statistically significant increase in two of the pregnancy risk factors: the proportion of women with a history of caesarean delivery increased from 3 to 5% after FANC and miscarriage increased from 11 to 13% after FANC. Lastly, there is also a significant increase in media access through radio and television in the years following FANC implementation.

### Early access to care

**Fig. 1:**
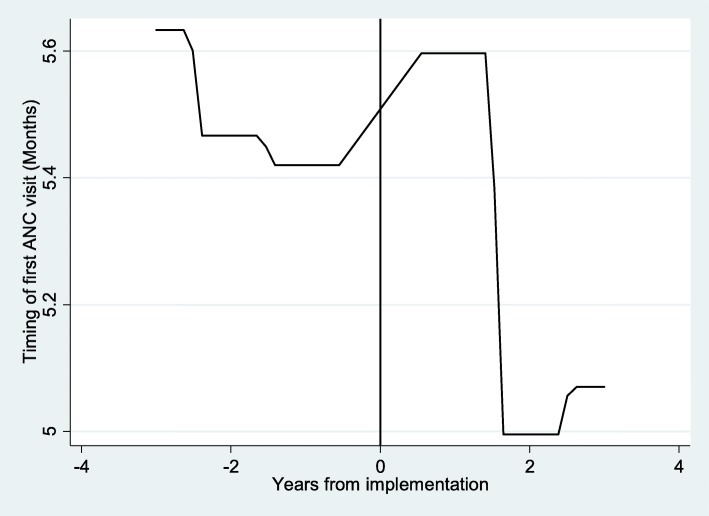
Timing of first antenatal care visit. Notes: lpoly smoother, no controls- X-axis; 0- represent 2003, the year of FANC implementation; vertical line demarcates the two periods; Negative years indicate number of years before FANC and positive years the number of years after FANC implementation. Y-axis-timing of first ANC visit (measured in months)

Figure 1, shows changes in the timing of the first ANC visit between pre- and post-FANC periods. The *y-axis* represents the month after conception during which a woman initiated her first ANC visit and the *x-axis* represents the number of years from the implementation of FANC. Negative (positive) values are the number of years before (after) FANC implementation, while zero indicates the time of implementation in 2003. Similar to the descriptive statistics, we notice a significant reduction in late access following FANC implementation, over and above the existing downward trend that emerged before FANC.

Table 4 presents the Weibull regression results for the relationship between FANC adoption and early access to care. We find that FANC had decreased the likelihood of early access when we look merely at the levels, but this effect is overwhelmed by the positive trend effect. It is encouraging to see that there is a positive coefficient on the post-FANC trend and that the implementation of this policy has increased early access.

**Table 4:** Impact of FANC on early access to care: Weibull model with interrupted time series analysis

Variables	Coefficient	Standard error (S.E)
FANC Policy dummy		
FANC	−0.370^a^	(0.086)
Time trend	0.018	(0.031)
Post FANC x Time Trend	0.207^a^	(0.036)
Woman’s education level (base = no educ)		
Primary	0.192^a^	(0.042)
Secondary	0.363^a^	(0.066)
Tertiary	0.463^b^	(0.201)
Pregnancy risky indicators		
16 years and below	−0.156	(0.141)
Above 40	−0.218^a^	(0.074)
Miscarriage	0.108^a^	(0.042)
Caesarean deliveries	0.126	(0.077)
Access to radio (Base = no access)		
Less than once a week	0.110^b^	(0.051)
At least once a week	0.078	(0.055)
Almost every day	0.204^a^	(0.044)
Access to television		
Less than once a week	−0.186	(0.061)
At least once a week	0.047	(0.109)
Almost every day	0.051	(0.077)
Community type		
Urban	0.110^b^	(0.046)
Community HIV-test statistics	0.105	(0.089)
Constant	−7.182^a^	(0.0125)
Observations	8522	
Wald Chi2(1)	299.92^a^	

Notes: Weibull survival models. ^a^1% level of significance; and ^b^ 5% level of significance. Coefficients with standard errors in parentheses

Furthermore, having at least primary-level education, access to radio, having been tested for HIV, a history of miscarriage or caesarean delivery, and urban residence are all associated with a higher likelihood of early access to care.

### Underutilisation of care

**Fig. 2:**
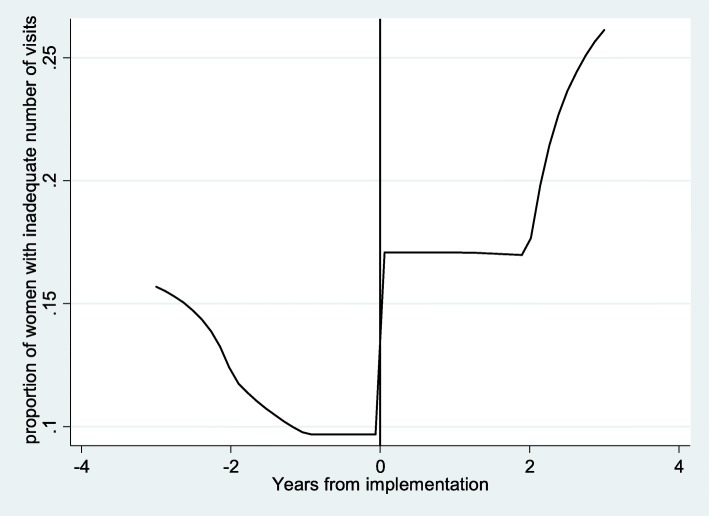
Change in underutilisation of ANC services in a cluster. Notes: lpoly smoother, no controls- *X-axis*; 0- represent 2003, the year of FANC implementation; vertical line demarcates the two periods; Negative years indicate number of years before FANC and positive years the number of years after FANC implementation. Y-axis-proportion of women with inadequate number of visits in a cluster

Figure 2, shows an increase in the community-level underutilisation of ANC services after FANC adoption. Table 5 confirms that FANC has had the unintentional consequence of increasing underutilisation. We see that before FANC, women were progressively less likely to underutilise ANC services over time. After FANC was adopted underutilisation increased by eight percentage points per year (after accounting for the confounding variables), relative to the initial downward trend.

**Table 5:** Impact of FANC on underutilisation of ANC services: OLS model with interrupted time series analysis

Variables	Coefficient	Standard error (S.E)
FANC Policy dummy		
FANC	0.034	(0.046)
Time trend	−0.028^b^	(0.014)
Post FANC x Time Trend	0.083^a^	(0.021)
Woman’s education level (Base = No Educ)		
Primary	0.016	(0.031)
Secondary	0.068	(0.044)
Tertiary	0.106	(0.144)
Media access (Base = No access)		
Radio		
Less than once a week	−0.081^c^	(0.042)
At least once a week	−0.103^b^	(0.044)
Almost every day	−0.038	(0.034)
Location		
Urban	−0.025	(0.027)
HIV_test	−0.038	(0.031)
Television	Yes	
Pregnancy risky indicators	Yes	
Constant	0.119^b^	(0.046)
Observations	1606	
R-squared	0.0406	

Notes: Ordinary Least Squares Regression. ^a^1% level of significance; ^b^ 5% level of significance and ^c^10% level of significance. Coefficients with standard errors are in parenthesis

Very few of the control variables and the proxies for confounding factors were statistically significant in these regressions. Those respondents who had access to the radio at least once a week are more likely to underutilise care.

### Quality of care

Table 6 reports descriptive results on individual level quality of care. The findings show an overall improvement in compliance of individual ANC contents after FANC. Women who reported to have received all eight components increased from 9 to 11% in the post FANC period. There are significant increases in taking of blood and samples, communication about complications, dispensing of iron tables and dispensing of malaria prophylaxis.

**Table 6:** Services received during antenatal care visit

Variable categories	Proportion of women		*p*-value
	Pre-FANC	Post-FANC	
Weighed during pregnancy	0.96	0.95	0.2396
Blood pressure checked during pregnancy	0.79	0.79	0.4546
Urine sample taken during pregnancy	0.20	0.22	0.0638^*^
Blood sample taken during pregnancy	0.37	0.47	0.0000^***^
Told about complications	0.69	0.72	0.0032^***^
Given or instructed to go buy iron tablets	0.77	0.84	0.0000^***^
Took Fansidar as prophylaxis for malaria	0.76	0.84	0.0000^***^
Received at least one tetanus injection	0.83	0.83	0.3319
Received all eight	0.09	0.11	0.0002^***^

^***^significant at 1% level of significance; and ^*^10% level of significance

**Fig. 3:**
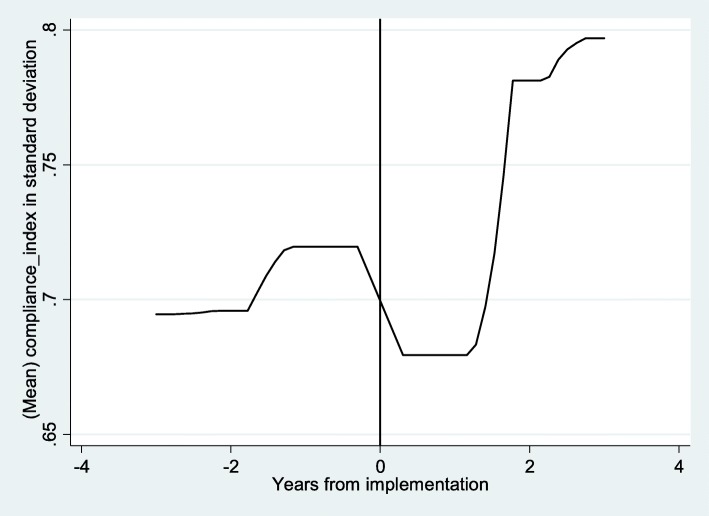
Change in quality of care. Notes: lpoly smoother, no controls- X-axis; 0- represent 2003, the year of FANC implementation; vertical line demarcates the two periods; Negative years indicate number of years before FANC and positive years the number of years after FANC implementation. Y-axis- cluster level mean compliance index in standard deviation

In Fig. 3, we show the change in overall quality of ANC services at the community level, as measured by the MCA index. In the years immediately after FANC implementation, there was a decline in quality, which was then followed by a period of stagnation before a substantial improvement. This could represent a one-year lag or initial teething problems with the implementation of this policy.

The coefficients reported in Table 7 show no evidence of FANC improving the quality of care. Based on the descriptive results we consider the hypothesis that there may have been teething problems with implementation or a lag in implementation, changing the policy cut-off point to 2004, but this specification also does not provide any evidence of an improvement in the quality of care.

**Table 7:** Impact of FANC on quality of ANC services: OLS model with interrupted time series analysis

Variables	Coefficient	Standard error (S.E)
FANC policy dummy		
FANC	−0.067^a^	(0.020)
Time trend	0.013^b^	(0.006)
Post FANC x Time trend	0.008	(0.009)
Woman’s education level (Base = No educ)		
Primary	0.042^a^	(0.013)
Secondary	0.096^a^	(0.018)
Tertiary	0.023	(0.062)
Community type		
Urban	0.032^a^	(0.011)
HIV-testing prevalence		
HIV-test statistics	0.086^a^	(0.013)
Pregnancy risk indicators		
16 years and below	−0.023	(0.043)
Above 40 years	0.031	(0.024)
Miscarriage	0.001	(0.015)
Caesarean	0.018	(0.023)
Type of health worker		
Doctor	0.043^b^	(0.020)
Nurse	0.062^a^	(0.021)
TBA	−0.324^a^	(0.033)
Ward attendant/ health surveillance assistants	−0.002	(0.023)
Constant	0.632^a^	(0.027)
Observations	1606	
R-Squared	0.2252	

NOTES: Ordinary Least Squares regressions. ^a^significant at 1% level of significance; and ^b^ significant at 5% level of significance. Coefficients with standard errors in parentheses

Urban location, education and HIV-testing prevalence rates are all positively associated with quality of care. In addition, skilled health providers (doctors and nurses) are more likely than unskilled providers (TBAs) to comply with the set of protocol measures in our quality index.

## Discussion

ANC provides a critical opportunity to support pregnant women and ensure that they, and their babies, benefit from effective, good-quality maternal care. This paper analysed the impact of the 2001 WHO Focused Antenatal Care model on quality and use of ANC services currently used in Malawi at a national level. Unlike previous studies, this study used nationally representative data and appropriate statistical methods to provide evidence on the impact of FANC in low resourced settings. Given the 2016 revision of ANC guidelines, this study is timely and important in order to evaluate the effectiveness of the FANC policy, which is still followed in Malawi.

### Early access to care

Our results show a positive association between FANC and early access. The findings demonstrate a significant increase of about 21 percentage points per year (relative to baseline) in early access after FANC adoption. FANC has helped to promote early access in Malawi. We find the results plausible because FANC required the first contact to occur by 16 weeks of pregnancy [2]. However, the descriptive results show that 3 years after FANC implementation only 29% of women in our sample initiated ANC by 16 weeks of pregnancy. At a national level, the MDHS reports that only 24% of women initiated ANC early in 2015 [35]. Many women delay access to care beyond the first trimester and this is worrying given that HIV transmission remains a concern.

Furthermore, our results show that women who have at least primary education are more likely to initiate their first ANC visit in the first trimester. This finding is similar to that of Yaya et al. [36] who argued that educated women are more knowledgeable about the importance of ANC visits. Similarly, earlier studies conducted by Furuta and Salway [37] in Nepal suggest that education promotes new values and attitudes that are favourable to the use of modern health care, thereby increasing the likelihoods of women accessing skilled care. Consequently, policies aimed at improving female education are likely to also increase ANC usage [28]. This is particularly important in Malawi, where only 1 in 4 women have at least some secondary education and only 3% of women have tertiary education [35].

Women with a history of miscarriage and/or caesarean deliveries are also more likely to initiate ANC early, confirming the results found by Pell et al. [38]. This finding is of particular importance because it shows that women are responding to FANC’s emphasis on complications and danger signs to watch for in pregnancy. Our study also finds that women who have access to a radio at least once a week are more likely to initiate their first ANC visit early. This finding is consistent with findings by Bbaale [39]. They concluded that it was necessary to focus on educating girls beyond the secondary level, but also to increase media penetration for the purpose of spreading information about the importance of ANC visits.

The type of residence a woman reports to come from has a significant impact on early access in our study. Women who reside in urban communities were more likely to initiate their first ANC visit early compared to women from rural communities; this finding is consistent with previous studies [40–42]. Supply factors such as the quality of ANC services provided and the distance of the healthcare facility have been reported to play a significant role [43]. Malawi has promoted FANC via free public provision, however, the health facility distribution favours urban areas [5].

Other factors influencing the timing of first ANC visits, as reported by qualitative studies in Malawi, include women’s beliefs [43] and user fees in CHAM (Christian association of Malawi) hospitals [3]. Evidence shows that mothers often seek care or pregnancy lessons from traditional health workers and elderly women before seeing medical personnel at a facility in Mangochi, Malawi [43]. This practice stems in part from the belief that it is not necessary to attend clinics or hospitals unless one is physically ill. Furthermore, while FANC health services are free at the point of use in public facilities, in communities where the majority of facilities are managed by CHAM, they pay user fees at the point of use of 2000 Kwacha [3 US$] on average to access FANC services [3]. Many rural and poor women in Malawi cannot afford the cost to access private FANC services.

### Underutilisation of care

The results show that FANC has been associated with unintended increases in underutilisation of ANC services. These results are consistent with one previous Zambian study where the researcher reported a significant decline in the number of visits to an average of three following FANC adoption [44]. A qualitative study in Malawi reported that some FANC requirements served as barriers to accessing and adequately utilising FANC services in Mangochi [3]. In particular, FANC encourages men to engage in reproductive health and preparation for birth. The study reports that most of the health facilities in the district have by-laws formulated by health officials and community leaders stating that a pregnant woman can only be attended to if her spouse accompanies her. This led to many women not utilising FANC services. Furthermore, due to the requirement for birth preparedness as suggested by FANC guidelines, health workers demand that pregnant women bring with them cloth wraps for the baby (traditional “chitenje”) at their initial FANC visit [3]. All these requirements reportedly make FANC services expensive and therefore less accessible.

Another interesting result is the negative significant coefficient between underutilisation and access to radio. These results are consistent with those found by Bbaale [39]. Media access, especially access to radio, has been shown to be instrumental in changing behaviour regarding maternal health services utilisation [36, 42].

### Quality of care

There was an increase in blood testing, but 3 years after the implementation of FANC only 47% of women received blood tests. This level of access is disappointingly low given that blood sample testing is one of the essential factors in reducing HIV transmission from the mother to the unborn child. Under reporting is a distinct possibility due to potential recall bias or the sensitive nature of this question.

Only 22% of women, who gave birth after FANC was implemented, reported having been given a urine test during the ANC visit. Lungu et al. [9] found that there were shortages of essential laboratory supplies at one urban clinic in Malawi, which made it difficult to conduct the essential laboratory tests. Nyarko et al. [7] reported similar findings in Ghana. Our results also show that only 11% of users in our sample received all ANC components after FANC implementation. In Nigeria, only 5% of users received the optimal level of care (all 10 components) after FANC adoption [27].

Our regression results show no significant improvement in the quality of ANC services even after accounting for a one-year lag in adequate implementation of the policy. This is similar to the experience in South Africa, where FANC did not improve performance in maternal health delivery [8]. A multi-country level study conducted in selected low and middle-income countries found very low compliance rates to FANC standards among women who went for their required four ANC visits [45]. The results of these studies show that low and middle-income countries struggled to implement FANC and as such, the policy did not translate into improved quality in ANC services.

Our results did however show a significant positive association between HIV-testing share and quality index, suggesting that PMTCT may have had an impact on quality. We also find that the type of health worker providing ANC plays a vital role in determining the quality of ANC received by women. Women were more likely to receive good quality ANC care if skilled providers (doctors or nurses) attended to them compared with those who were relatively less skilled (patient attendant/ health surveillance assistants and traditional birth attendants). Similar results were also reported in Nigeria [27] and Nepal [28]. However, a qualitative study analysing women’s perspectives on quality of care in Mzimba, Malawi reported that health worker attitudes towards clients had negative impacts on the clients’ perceptions of quality received [46]. As such, women preferred traditional birth attendants over skilled personnel.

The average education level of mothers is also positively associated with higher-quality ANC services. As previously explained, educated mothers are more aware of their rights and more knowledgeable about health education and what is expected at the ANC clinics. Similar findings were reported in Nigeria [27], Nepal [28] and Uganda [39]. In line with previous studies, we find urban communities have better quality ANC services in comparison to rural-based clinics. A study in Nigeria, also reports similar results [27], where women in urban areas had higher odds of receiving good quality ANC compared to those in rural areas. This may be because in most cases, urban clinics are better endowed in terms of resources and budget allocations [5]. There is therefore a need to improve the quality of ANC services delivery in rural areas.

## Strengths and limitations of the study

The interpretation of these findings needs to be tempered by the limitations of the methodologies employed. First, the data used in the analysis were self-reported and given by respondents about events retrospectively, making the information collected subject to recall bias. An effort was made to reduce this by analysing data on the most recent pregnancy within 5 years of each survey and limiting the sample size to deliveries that happened 3 years before and after FANC adoption. Second, most of the variables (education, exposure to radio and television, HIV testing) were measured at the time of the survey rather than at the time of the birth.

Despite these limitations, this paper has a major strength. The study makes use of nationally representative data to estimate the impact of FANC on ANC quality and utilisation in a low resourced setting. Moreover, the statistical methods were able to more accurately assess the impact of FANC than the methods employed by previous studies in this research area.

## Conclusion

In Malawi, the implementation of FANC has been associated with increased early access; however, it is also associated with unintended consequences of underutilisation of ANC services and no change in the overall quality of maternal health services.

The successful implementation of FANC required investment in clinical skills, medical supplies and equipment [9]. In under-resourced health systems, this can pose a challenge. As reported in the 2010 annual monitoring report, only one of the four central hospitals and four of the 24 district hospitals in Malawi met the WHO standards for delivering FANC [16]. Policies that target strengthening the capacity of the health systems to implement FANC guidelines effectively would help to improve the quality and utilisation of ANC services. Policy makers can also explore whether further gains can be made by intensifying direct community campaigns and engagement through health surveillance assistants (HSAs) especially in rural areas where the results show relatively poor outcomes.

These recommendations apply more widely to health policy reforms in resource-constrained settings. Given that we find underutilisation when the benchmark is set at four visits, eight visits are unlikely to be feasible in settings that are struggling to comply with far less ambitious targets.

## Abbreviations

AIDS: Acquired immune deficiency syndrome
ANC: Antenatal care
CHAM: Christian association of Malawi
DHS: Demographic health survey
FANC: Focused Antenatal Care
FPE: Free primary education
GDP: Gross domestic product
HIV: Human immunodeficiency virus
ITSA: Interrupted time series analysis
MCA: Multiple correspondence analysis
MDHS: Malawi Demographic and Health Survey
OLS: Ordinary least squares
PMTCT: Prevention of mother-to-child transmission
TBA: Traditional birth attendant
TTV: Tetanus vaccine
US$: United states dollar
WHO: World Health Organization

## Footnotes

1 The second Malawi household integrated survey estimated that between 2004 and 2005, only 3.16% of the households in Malawi and 3.52% of women of reproductive age had access to mobile phones [31].
